# Supplementary material for: Smectic phase in suspensions of gapped DNA duplexes
Source: Nat Commun. 2016 Nov 15;7:13358. doi: 10.1038/ncomms13358 (PMC5116068; doi:10.1038/ncomms13358)
Supplement: Supplementary Information — Supplementary Figures 1-11, Supplementary Notes 1-6, Supplementary Methods and Supplementary References [file ncomms13358-s1.pdf]

Supplementary Figures

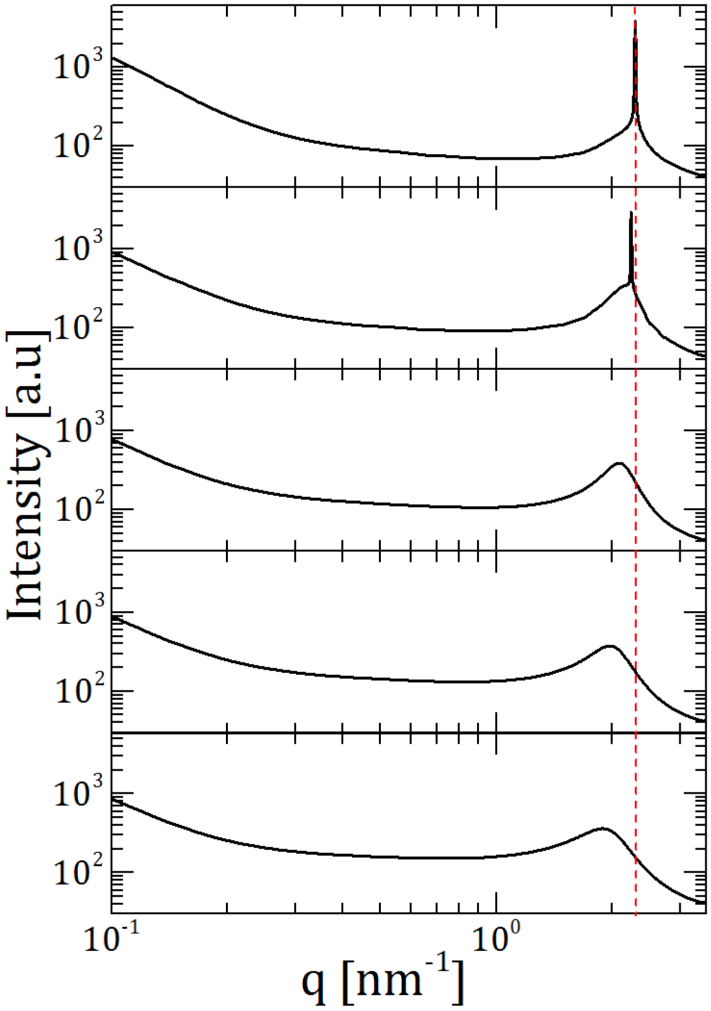

Supplementary Figure 1. 1D-SAXS profiles for the G_{1T}-duplex, where concentrations from top to the bottom are 295, 285, 273.3, 249.4, 237.2 mg ml^{-1}. The dotted line is a guide for the concentration dependence of the peaks position at scattering wave vectors q>0.1nm^{-1}.

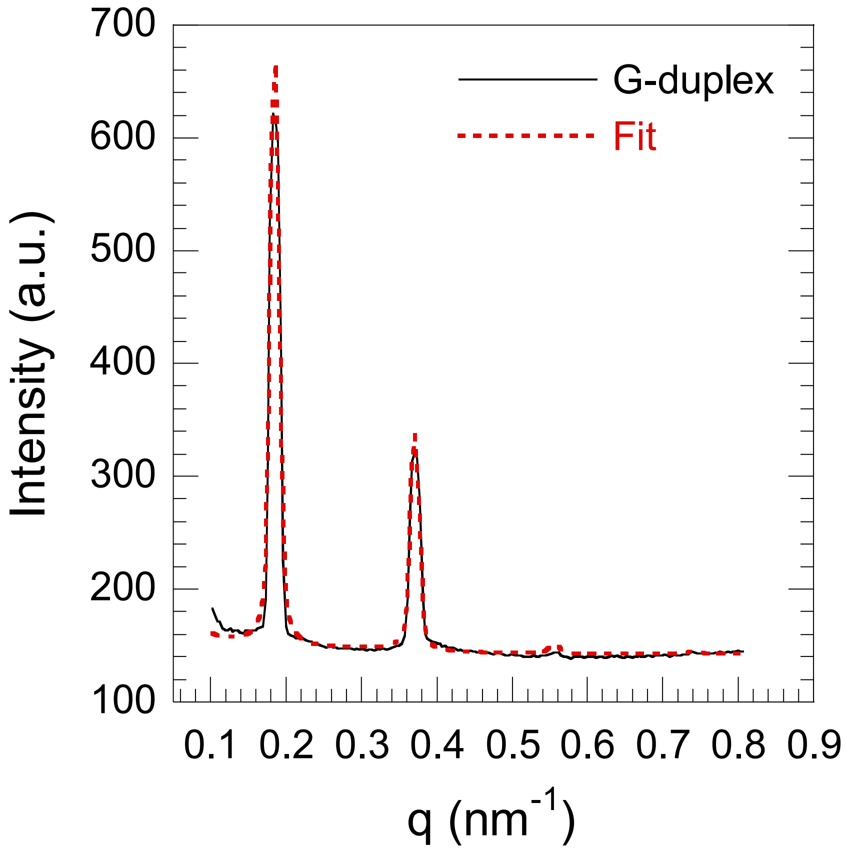

Supplementary Figure 2. 1D-SAXS profile for G_{20T}-duplex at a concentration of 242 mg ml^{-1}, and a fit of the function (1).

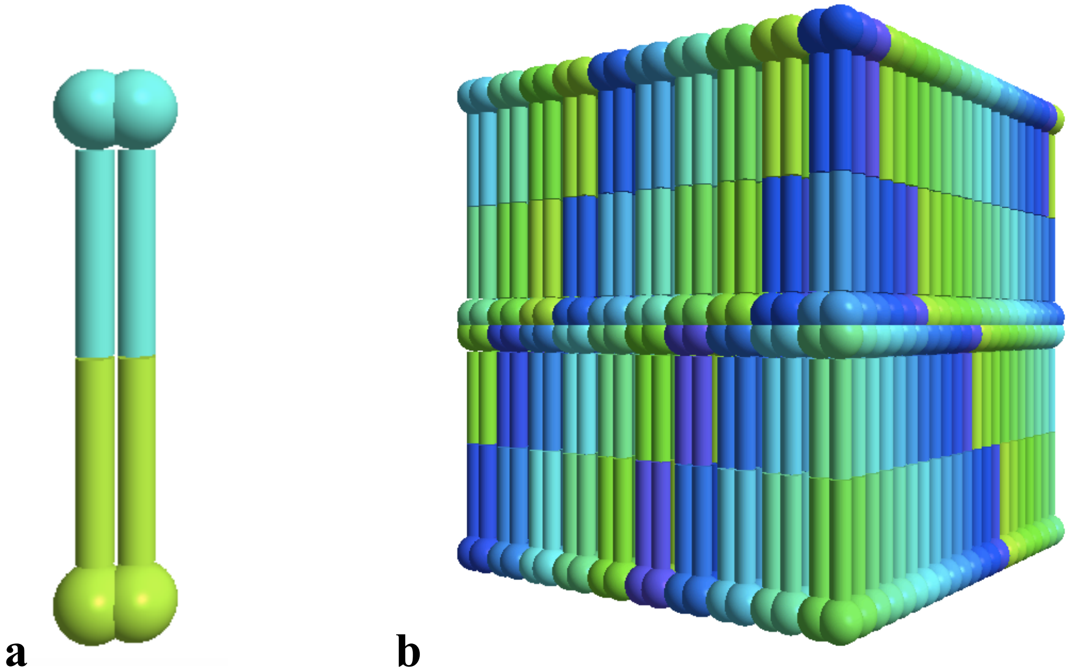

Supplementary Figure 3. Initial configuration for equilibration (**b**) built from dimers of G-duplexes in a fully folded configuration (**a**).

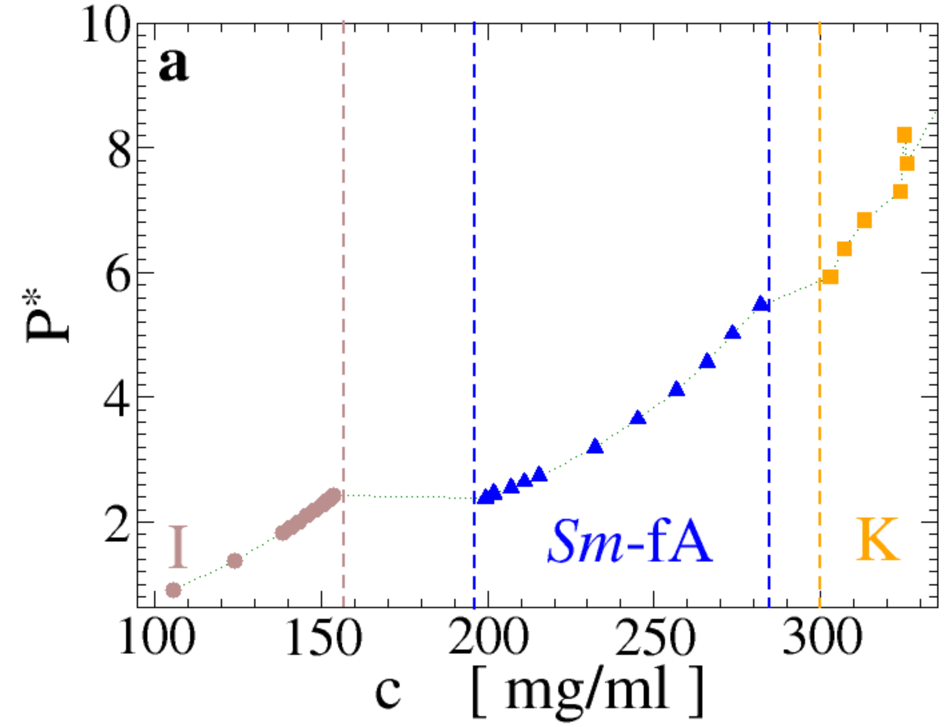

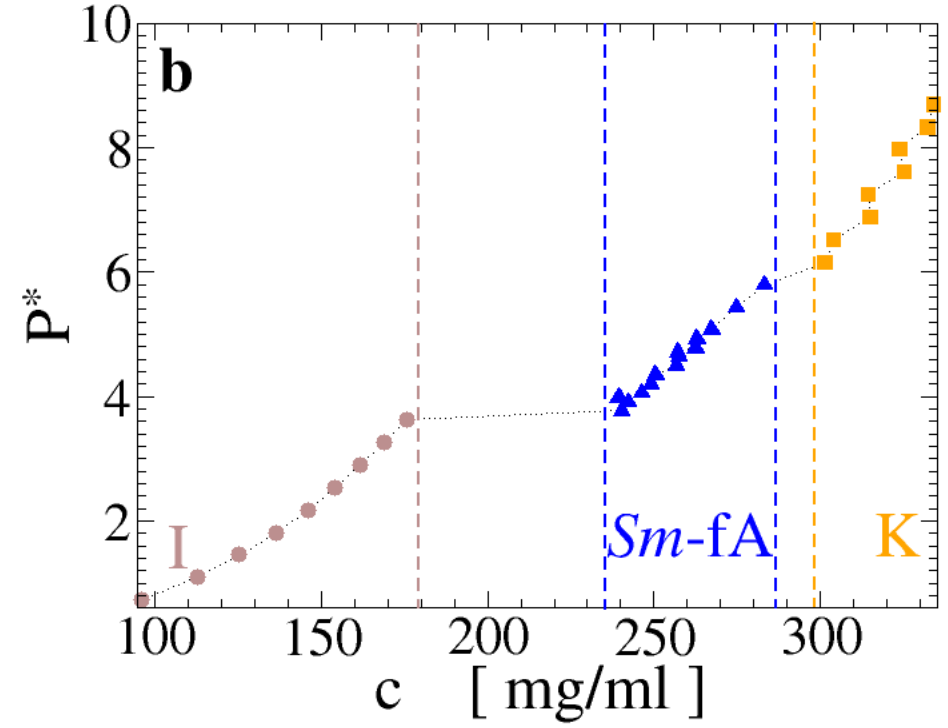

Supplementary Figure 4. Equation of state for (**a**) $\beta u_0 = 8.06$ and (**b**) $\beta u_0 = 6.41$.

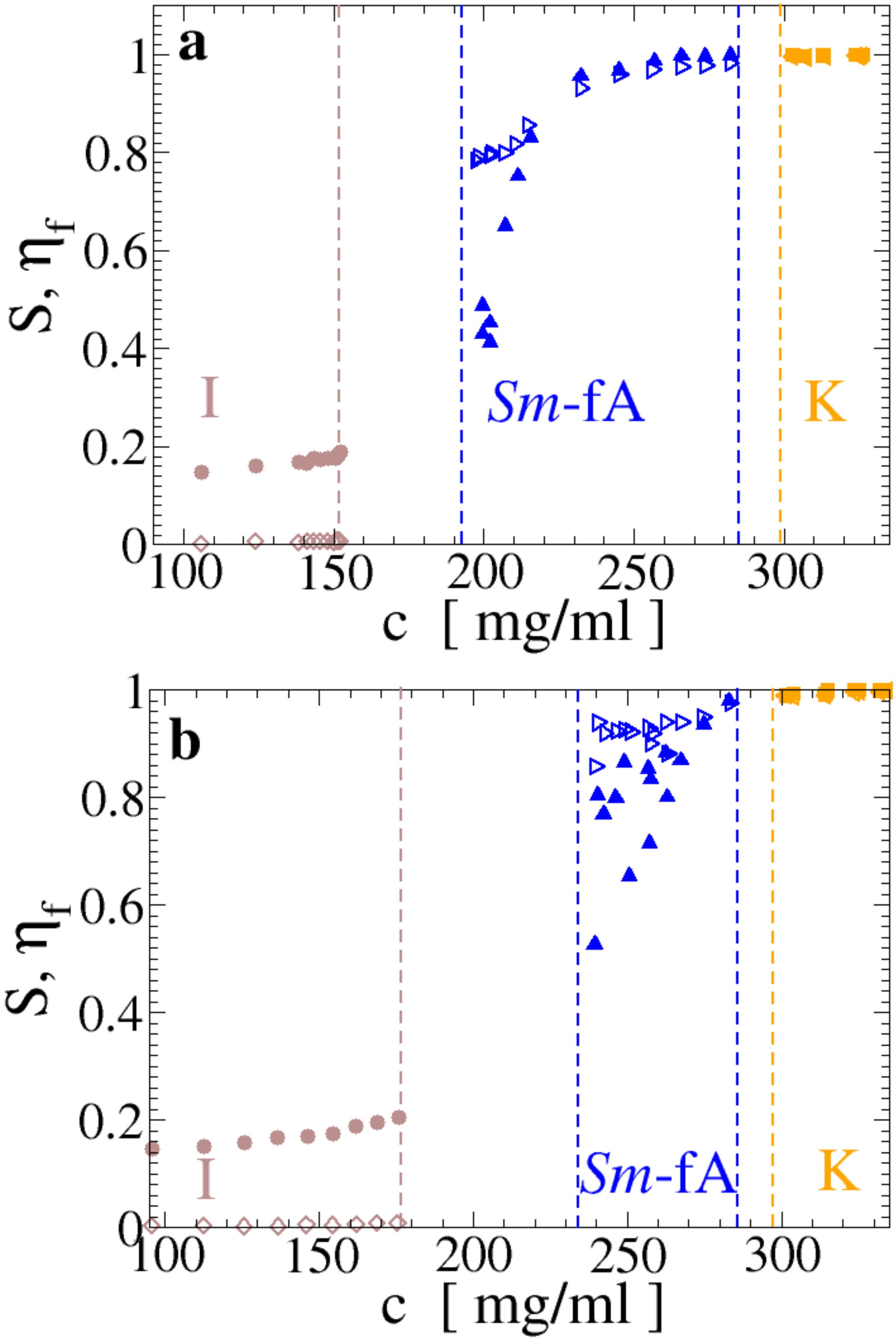

Supplementary Figure 5. Fraction of folded G-duplexes (filled symbols) and nematic order parameter S (open symbols) for (**a**) $\beta u_0 = 8.06$ and (**b**) $\beta u_0 = 6.41$.

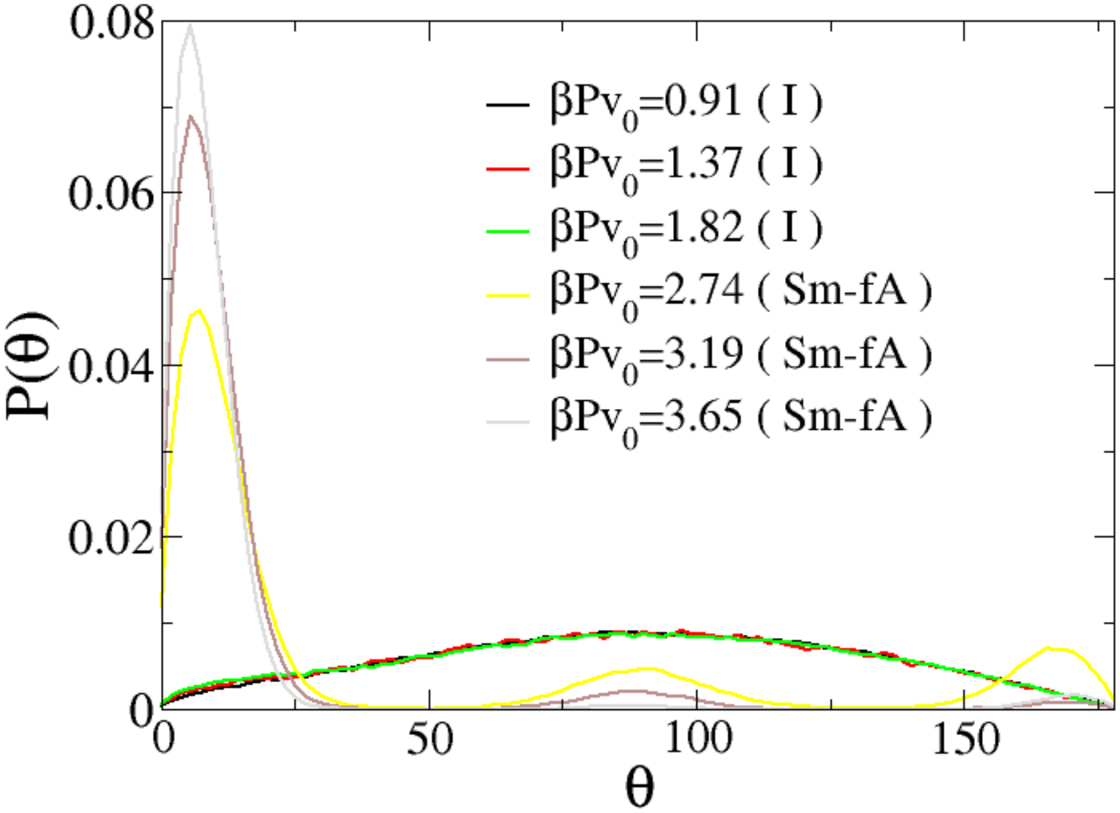

Supplementary Figure 6. Distribution of the angle between the two cylinders belonging to the same gapped duplex for different pressures for isotropic ($\beta P v_0 = 0.91, 1.37$ and 1.82), and smectic states ($\beta P v_0 = 2.74, 3.19$ and 3.65).

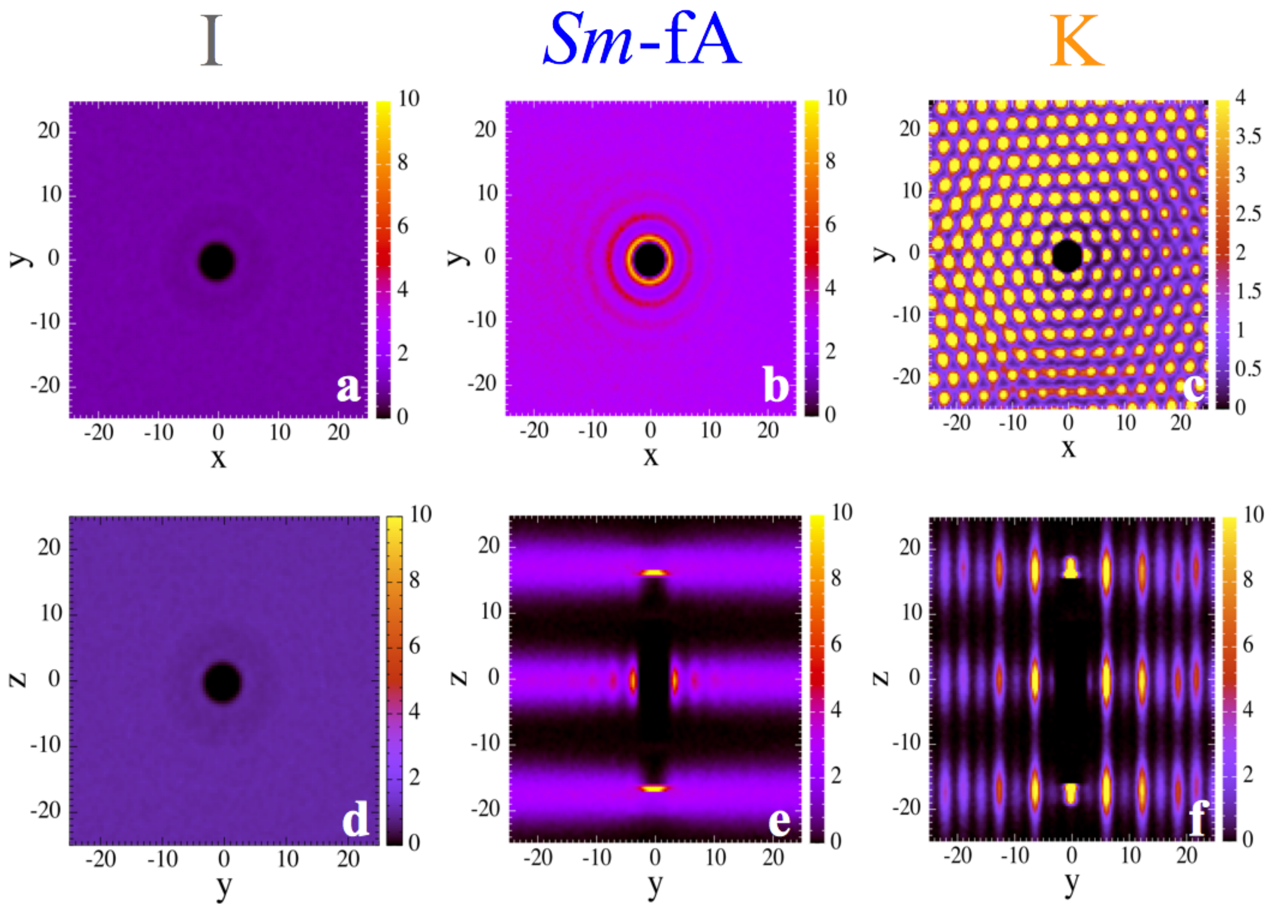

Supplementary Figure 7. Pair distribution function on a plane perpendicular ($g(x,y,0)$ in panels (**a-c**)) and parallel ($g(0,y,z)$ in panels (**d-f**)) to the nematic director for $\beta u_0 = 8.06$ for selected pressure to show typical patterns for the isotropic (*I*), folded smectic-A (*Sm*-fA) and crystal (*K*) phases. (**a**) and (**d**) P^*=2.3; (**b**) and (**e**) P^*=4.1; (**c**) and (**f**) P^*=6.4.

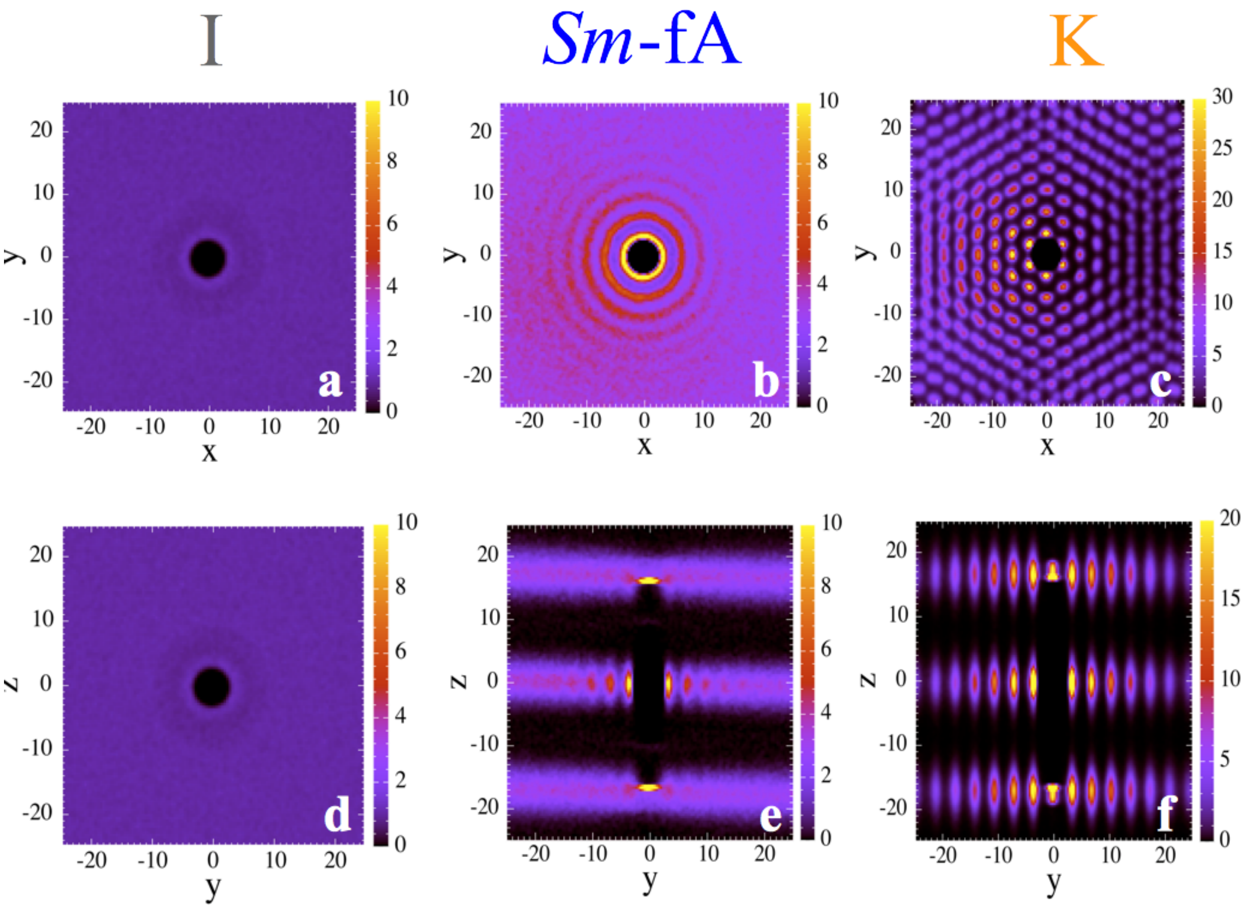

Supplementary Figure 8. Pair distribution function on a plane perpendicular ($g(x,y,0)$ in panels (**a-c**)) and parallel ($g(0,y,z)$ in panels (**d-f**)) to the nematic director for $\beta u_0 = 6.41$ for selected pressure to show the different patterns obtained in the isotropic (*I*), folded smectic-A (*Sm*-fA) and crystal (*K*) phases. (**a**) and (**d**) P^*=3.6 ; (**b**) and (**e**) P^*=5.8; (**c**) and (**f**) P^*=6.9.

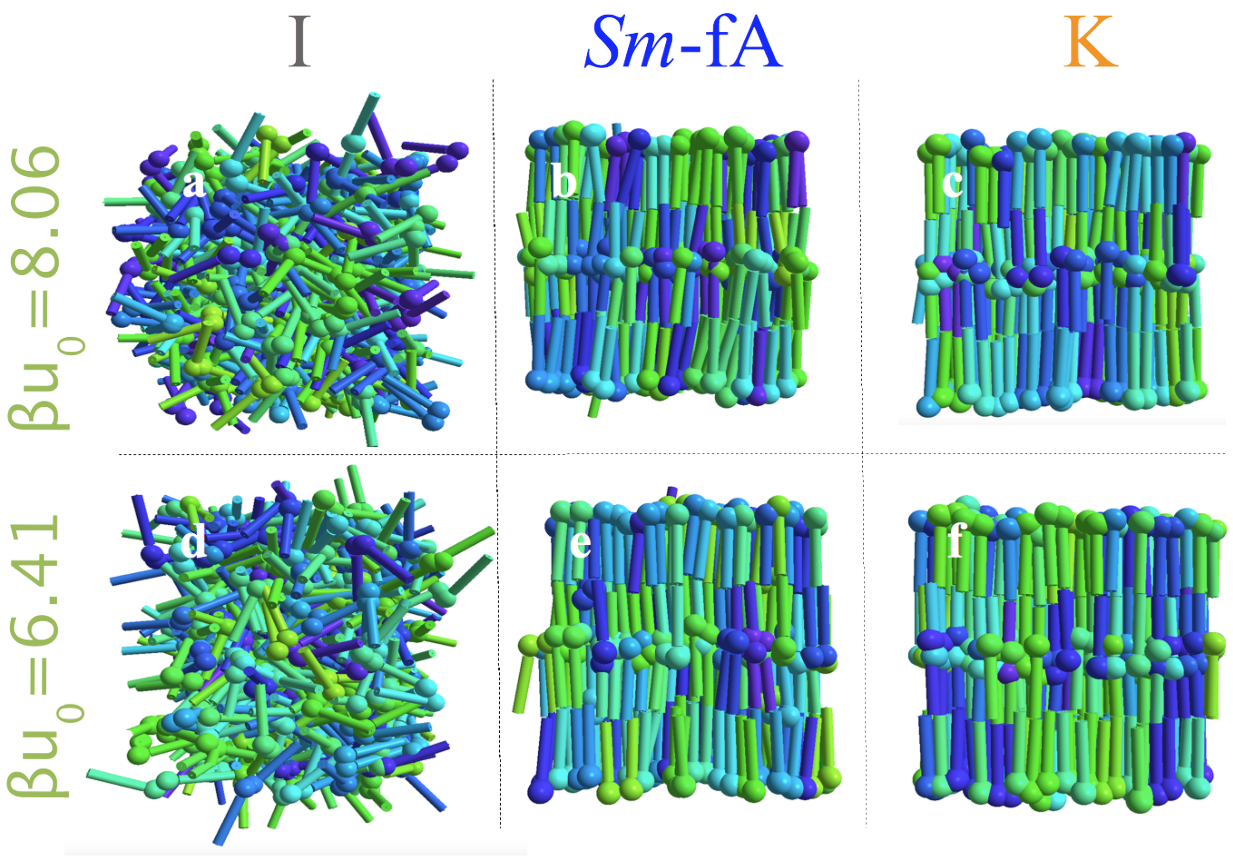

Supplementary Figure 9. Snapshots of the different phases obtained in the simulations for $\beta u_0 = 8.06$ (**a-c**) and $\beta u_0 = 6.41$ (**e-f**). (**a**) P^*=2.3, (**b**) P^*=4.1, (**c**) P^*=6.4, (**d**) P^*=3.6 (**e**), P^*=5.8, (**f**) P^*=6.9.

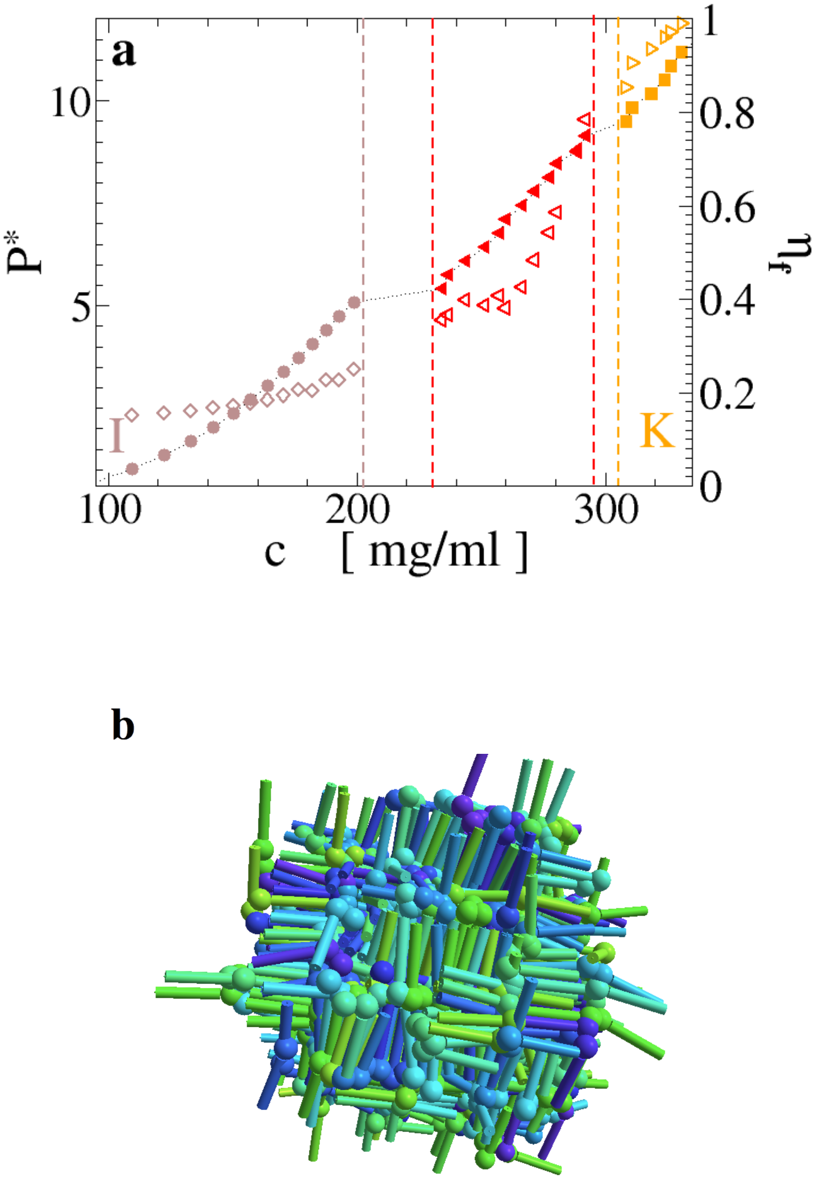

Supplementary Figure 10. (**a**) Equation of state (filled symbols) and fraction of folded particles (open symbols) for $\beta u_0 = 0$. (**b**) Snapshot for $P^* = 6.1$ of the partially smectic-A phase observed in this case between the isotropic phase (I) and the crystal (K).

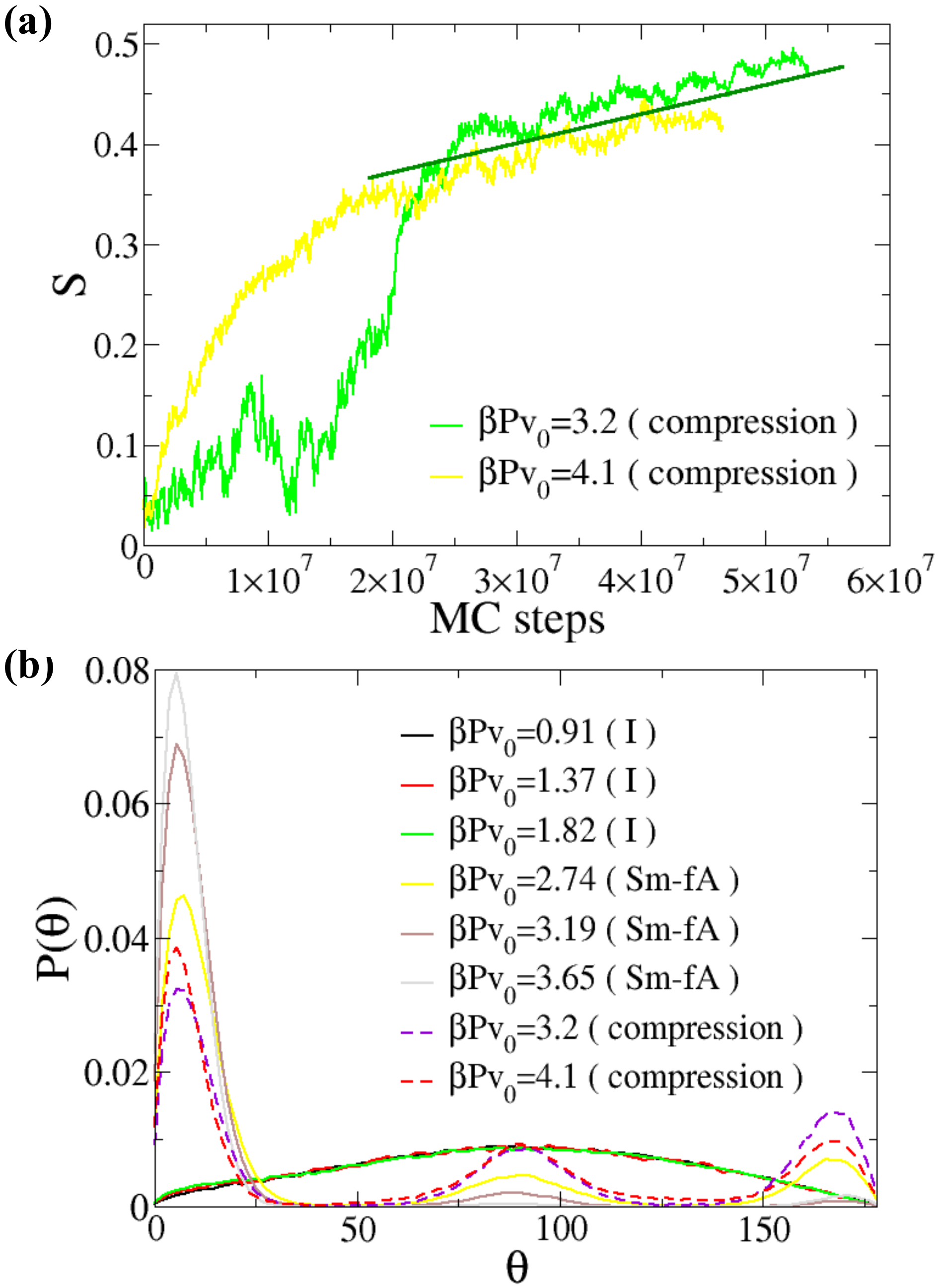

Supplementary Figure 11. **(a)** Order parameter as a function of MC steps for the two-equilibration runs at pressures $\beta P v_0 = 3.2$ and 4.1. The straight line is just a guide to the eye, which evidences the ongoing equilibration process. (**b**) Distribution of the angle between the two cylinders belonging to the same gapped duplex for different pressures. The two curves labeled as "compression" have been obtained from equilibration runs with a hydrophobic attraction $\beta P v_0 = 8.06$ starting from an initial isotropic configuration at $\beta P v_0 = 3.2$ and 4.1

Supplementary Notes

Contents

1. **SAXS measurements on concentrated solutions of G_{1T}-duplex**
2. **Electron density profile reconstruction**
3. **Simulations with hydrophobic interactions**
4. **Excluded volume calculations in the Smectic phase**
5. **Simulations without hydrophobic interactions**
6. **Equilibration-runs from the isotropic phase**

Supplementary Note 1: SAXS measurements on concentrated solutions of G_{1T}-duplex

1D-SAXS profiles at room temperature for the G_{1T}-duplex for various concentrations above the I/LC coexistence region are shown in Supplementary Fig. 1. It is clear that the SAXS patterns do not exhibit any evidence of smectic ordering up to 300 mg ml^{-1}.

Supplementary Note 2: Electron density profile reconstruction

The *Inverse Fourier Transformation* method was employed for the calculation of the electron density profile from the 1D-SAXS profiles. The details are given below.

The measured scattering intensity is proportional to the product of the form factor $|F(q)|^2$ and the structure factor $S(q)$

$$I(q) \propto |F(q)|^2 S(q) \qquad (1)$$

For a 1D lattice constituted by *n* electron density levels of thickness *d*, the scattered amplitude has the form

$$F(q) = \sum_{i=1}^{n-1} \frac{2(\rho_{i+1} - \rho_i)}{q} \sin\left(\frac{q d_i}{2}\right) \qquad (2)$$

The electron density profile *r*(z) can be calculated by inverse cosine Fourier transform from the scattering amplitude $F(q)$ as

$$\rho(z) = 2\int_0^{\infty} F(q)\cos(qz)\, e^{-0.5q^2} dq \tag{3}$$

The structure factor that modeled best our data is a 1D lattice with thermal disorder [1, 2].

The best fit to the experimental data was obtained by using a model with two electron density levels (Supplementary Fig. 2). The addition of more electron density levels in the model did not further improve the fit.

The width of the diffraction peaks is related to the average coherent domain size in the direction perpendicular to the smectic-fA layers z, and is accounted in the structure factor used. For the G-duplex the average coherent domain size is calculated to be around 350 nm.

Supplementary Note 3: Simulations with hydrophobic interactions

Equation of state for $\beta u_0 = 8.06$ and 6.41 is shown in Supplementary Fig. 4. Three phases can be easily identified, namely the isotropic phase (*I*), the folded smectic-A phase (*Sm*-fA) and the crystal (*K*). These phases have been characterized using the pair distribution function (Supplementary Figs 7,8), snapshots (Supplementary Fig. 9), the order parameter S (Supplementary Fig. 5) and the fraction of folded G-duplexes η_{f} (Supplementary Fig. 5). We start observing that *Sm*-fA and *K* are liquid crystalline phase as it can be deduced by the nematic order parameter shown in Supplementary Fig. 5. In addition, *Sm*-fA phase exhibits a significant fraction of folded G-duplexes (Supplementary Fig. 5): in the *I* phase the fraction of folded duplexes is around the value proper of a random distribution of orientations, while in the *Sm*-fA it jumps to values close to 0.5 and a fully folded system is observed in the crystal *K*. The distribution of folding angles for several isotropic and smectic state points is shown in Supplementary Fig. 6. It can be seen that in the *Sm*-fA phase the overwhelming number of duplexes attains a folded configuration and this number increases on increasing the pressure.

Finally we observe that from the equation of state it can be seen that on reducing the strength of the hydrophobic interaction (i.e. on increasing the temperature) the concentration of the *Sm*-fA at coexistence increases. Eventually at high temperatures the *Sm*-fA is destabilized (see **Supplementary Note 5: Simulations without hydrophobic interactions** below). Pair

distribution function shown in Supplementary Figs 7,8 reveals that the *K* phase is a hexagonal columnar crystal as it can be also concluded from snapshots shown in Supplementary Figs 9c, 9f.

Supplementary Note 4: Excluded volume calculations in the Smectic phase

In the smectic A phase with the nematic director along the z-axis, the excluded volume of two anisotropic particles can be written as follows [7]:

$$v_{\mathrm{excl}} = -\frac{1}{V}\int d\mathbf{R}_1\, d\mathbf{R}_2 d\mathbf{\Omega}_1 d\mathbf{\Omega}_2 e_{12}(\mathbf{R}_{12}, \mathbf{\Omega}_1, \mathbf{\Omega}_2) f_l(z_1, \mathbf{\Omega}_1) f_{l'}(z_2, \mathbf{\Omega}_2) \tag{4}$$

where $\mathbf{R}_1 = (x_1, y_1, z_1)$ and $\mathbf{R}_2 = (x_2, y_2, z_2)$ are the positions of the two particles, $\mathbf{R}_{12} = \mathbf{R}_1 - \mathbf{R}_2$, $\mathbf{\Omega}_1$ and $\mathbf{\Omega}_1$ are their orientations and

$$e_{12}(\mathbf{R}_{12}, \mathbf{\Omega}_1, \mathbf{\Omega}_2) = exp[-U_{\mathrm{h}}(\mathbf{R}_{12}, \mathbf{\Omega}_1, \mathbf{\Omega}_2)/k_{\mathrm{B}}T] - 1 \tag{5}$$

is the Mayer function with U_{h} being the interaction potential between the two particles.

For two gapped duplexes either in a fully unfolded or fully folded configuration we calculated numerically the excluded volume v_{excl} and v'_{excl} respectively by using a Monte Carlo integration. The numerical procedure is identical to the one discussed in Supplementary ref. 8, except that in the present case the centers of mass of the two gapped duplexes are constrained to be onto the plane $z = 0$ to account in a simplified way for the smectic layering in the system. From our numerical calculation we obtain that: $v_{\mathrm{excl}}/v'_{\mathrm{excl}} \approx 1.4$.

Supplementary Note 5: Simulations without hydrophobic interactions

Suppressing the hydrophobic interaction between the G-duplexes destabilizes the *Sm*-fA phase and the system exhibits a transition from an isotropic to a phase characterized by the presence of single layers with a limited extent, which do not regularly stack as in the *Sm*-fA. Layers in this phase are placed at right angles and this behavior arises from the presence in the system of a significant number of duplexes in right angle conformation due to the absence of stabilizing hydrophobic attractions. Nevertheless, we observe that in this intermediate phase a significant

fraction of fully folded particles is still present as shown in Supplementary Fig. 10. A further characterization of this mesophase will be provided in a future publication.

Supplementary Note 6: Equilibration-runs from the isotropic phase

To address the thermodynamic stability of the smectic-fA phase, we carried out MC simulations starting from an isotropic phase with $\beta u_0 = 8.06$ (as in the simulations show in the manuscript) at two different pressures. Although a proper equilibration is not achieved despite the very long simulation timespan (about 5×10^7 MC steps over 3 months), due to the slowness of folding kinetics, we find evidence of a partially folded phase (with about 20% of fully unfolded duplexes) with an order parameter $S \sim 0.5$. As shown below in Supplementary Fig. 11a, the order parameter, plotted as a function of MC steps, shows a clear trend towards greater values on further equilibrating the system. Nevertheless, the distribution of the angle formed by the two cylinders belonging to the same duplex, which is shown in Supplementary Fig. 11b, already shows a remarkable resemblance with the one obtained at equilibrium for the *Sm*-fA phase, where the majority of the duplexes are in a folded conformation.

Supplementary Methods

Contents

1. DNA sequences

2. Monte Carlo Simulations

Supplementary Method 1: DNA sequences

The DNA strand sequences for the F-duplex and the G-duplexes used here are given below from 5' to 3' end. The G-duplexes contain three DNA single strands, which fall into two groups: one long strand (strand 1) and two identical strands (strand 2 and 3).

F-duplex

Strand 1: ACA GAT GCA CAT ATC GAG GTG GAC ATC ACT TAC GCT GAG TAC TTC GAA TTT TTT TTT TTT TTT TTT TTA CAG ATG CAC ATA TCG AGG TGG ACA TCA CTT ACG CTG AGT ACT TCG AA

Strand 2: TTC GAA GTA CTC AGC GTA AGT GAT GTC CAC CTC GAT ATG TGC ATC TGT AAA AAA AAA AAA AAA AAA AAT TCG AAG TAC TCA GCG TAA GTG ATG TCC ACC TCG ATA TGT GCA TCT GT

G_{1T}-duplex

Strand 1: ACA GAT GCA CAT ATC GAG GTG GAC ATC ACT TAC GCT GAG TAC TTC GAA TAC AGA TGC ACA TAT CGA GGT GGA CAT CAC TTA CGC TGA GTA CTT CGA A

Strand 2: TTC GAA GTA CTC AGC GTA AGT GAT GTC CAC CTC GAT ATG TGC ATC TGT

Strand 3: TTC GAA GTA CTC AGC GTA AGT GAT GTC CAC CTC GAT ATG TGC ATC TGT

G_{20T}-duplex

Strand 1: ACA GAT GCA CAT ATC GAG GTG GAC ATC ACT TAC GCT GAG TAC TTC GAA TTT TTT TTT TTT TTT TTT TTA CAG ATG CAC ATA TCG AGG TGG ACA TCA CTT ACG CTG AGT ACT TCG AA

Strand 2: TTC GAA GTA CTC AGC GTA AGT GAT GTC CAC CTC GAT ATG TGC ATC TGT

Strand 3: TTC GAA GTA CTC AGC GTA AGT GAT GTC CAC CTC GAT ATG TGC ATC TGT

Supplementary Method 2: Monte Carlo Simulations

We carried out Monte Carlo (MC) simulations of N=840 G-duplexes modeled as two cylinders held together by a permanent bond between two patches located on their bases (see main text Fig. 4a for an illustration of the model). The algorithm to check the overlap between two cylinders has been adapted from Supplementary ref. 3, where we made some changes to increase its stability and efficiency. Speedup of equilibration has been achieved employing a cluster NPT algorithm adapted from Supplementary ref. 4. To model hydrophobic attraction between two G-duplexes we further decorate the cylinders with two patches on their bases and we performed NPT (isobaric) simulations at different pressures for $\beta P v_0 = 8.06, 6.41$ and 0. Note that the latter case amounts to not having hydrophobic interactions between the G-duplexes. The pressure will be given in reduced units, where the reduced pressure is defined as $P^* = \beta P v_0$, v_0 being the volume occupied by a single cylinder. To prepare the initial configuration for the equilibration stage we first create a dimer composed of two fully folded G-duplexes as shown in Supplementray Fig. 3a. Then we place N_d=N/2=420 of these dimers on a regular lattice as shown in Supplementray Fig. 3b. Following Supplementary refs 5,6 to build latter lattice we started from a close packed cubic lattice of hard spheres, we scaled the box along the z-direction by the length of a dimer and we place the dimers in a completely aligned configuration (i.e. with the nematic order parameter $S \sim 1$). After that we expanded the simulation box to achieve the desired initial volume fraction φ_0. We explored reduced pressures P^* ranging roughly from 1 to 10 and we chose values of φ_0 between 0.1 and 0.6 assuming a linear dependence on P^*. In our simulations we used usual periodic boundary conditions and minimum image conventions and in our isobaric simulation the box is allowed to change its size indepedently along the three directions x, y and z.

Supplementary References

1. Hosemann, R. & Bagchi, S. N. Direct analysis of diffraction by matter. xxi+734 (North-Holland Pub. Co., 1962).
2. Pabst, G. et al. Structural analysis of weakly ordered membrane stacks. *J. Appl. Crystallogr.* **36**, 1378–1388 (2003).
3. Ibarra-Avalos, N., Gil-Villegas, A. & Richa, A. M. Excluded volume of hard cylinders of variable aspect ratio. *Mol. Simul.* **33**, 505–515 (2007).
4. Almarza, N. G. A cluster algorithm for Monte Carlo simulation at constant pressure. *J. Chem. Phys.* **130,** 184106–184108 (2009).
5. Frenkel, D. Structure of hard-core models for liquid crystals. *J. Phys. Chem.* **92**, 3280-3284 (1988).
6. McGrother, S. C., Williamson, D. C. & Jackson, G. A re-examination of the phase diagram of hard spherocylinders. *J. Chem. Phys.* **104**, 6765–6771 (1996).
7. Wessels, P. P. F. and Mulder, B. M. Soft Mat. Nematic Homopolymers: From Segmented to Wormlike Chains. *Soft Mat.* **1**, 313-342 (2003).
8. De Michele, C. et al. Self-assembly of bifunctional patchy particles with anisotropic shape into polymer chains: Theory, simulations, and experiments. *Macromolecules* **45**, 1090-1116 (2012).
